# Participant Engagement in Supported Employment: A Systematic Scoping Review

**DOI:** 10.1007/s10926-021-09987-2

**Published:** 2021-06-04

**Authors:** Mariya Khoronzhevych, Tatiana Maximova-Mentzoni, Erika Gubrium, Ashley Elizabeth Muller

**Affiliations:** 1 Department of Social Work, Child Welfare and Social Policy, Oslo Metropolitan University, Pb. 4, St. Olavs plass, 0130 Oslo, Norway; 2 The Work Research Institute, Oslo Metropolitan University, Oslo, Norway; 3 Norwegian Institute of Public Health, Oslo, Norway

**Keywords:** Supported employment, Engagement, Vocational rehabilitation, Person-centred, Empowerment

## Abstract

*Purpose* This study aimed to synthesise the available knowledge on how participant engagement in supported employment (SE) interventions is presented, defined, and conceptualised. We also aimed to develop a working definition of participant engagement in SE based on the results of our study. *Methods* This systematic scoping review was conducted following the PRISMA extension for scoping reviews. The following databases were systematically searched: EBSCO, SCOPUS, Social Care Online, and JSTOR. We included peer-reviewed publications in English based on empirical studies. *Results* Sixteen articles met the inclusion criteria and were included in the final analysis. Thematic framework analysis resulted in three themes conveying the concept of participant engagement: self-determined choice, empowerment, and collaboration/working alliance. We suggest that participant engagement in SE is an active multifaceted process that involves the empowerment of participants, participants’ exercise of self-determined informed choice, and their collaboration with SE practitioners in a working alliance. *Conclusions* Participant empowerment, self-determined choice, and collaboration are important aspects of participant engagement in SE. The study results will appeal to SE practitioners and make significant contributions to the broader field of other vocational services supporting people in (re-)entering the competitive labour market.

**Supplementary Information:**

The online version contains supplementary material available at 10.1007/s10926-021-09987-2.

## Introduction

Previous research has shown that individualised rehabilitation intervention leads to better outcomes for service participants than do more broadly targeted rehabilitation measures [1, 2]. Individualisation of intervention heavily depends on participants’ engagement in the service and adjusting the intervention according to customised needs and wishes [3, 4]. Williams et al. highlight that engagement in rehabilitation is ‘a crucial patient characteristic in successful rehabilitation outcomes’ [4 p.1], while King et al. point out that it is ‘essential to mobilizing motivation and achieving desired change’ [5 p.2]. Low engagement is associated with low motivation, poorer therapeutic results, and longer rehabilitation time [4]. Engagement is important throughout all the multifaceted rehabilitation activities, including those performed at home between rehabilitation sessions [4, 5], those activities that are ‘personally valued’ [6], including social interactions and daily occupations [7], and in the pursuit of vocational goals [8].

In the context of vocational rehabilitation, engagement can take the form of participant collaboration, taking initiative, frequency of meetings with the counsellor adjusted to participants’ needs [9], intrinsic motivation, participants’ self-efficacy [10, 11], and the development of a ‘therapeutic’ relationship between the counsellor and the participant [12, 13]. Creating a working alliance with a participant appears to be critical for participant engagement, as it increases motivation and outcome expectancy [9, 10]. The participant may feel confident in providing feedback, and the provider consequently better able to provide the service accordingly, allowing for further service individualisation [14]. Given the significance of participant engagement in determining the success of rehabilitation interventions, researchers attempt to define and operationalise the concept of ‘engagement’ for measurement and implementation in practice [3, 15].

Supported employment (SE) is a type of vocational rehabilitation intervention that employs individualised support to improve employment outcomes in the ordinary labour market for people with various forms of disability. It originated in the United States in the late 1970s as a vocational rehabilitation intervention targeted at people with intellectual disabilities, people with mental disorders, and others labelled as having ‘the most significant disabilities’, to help them to obtain employment in a competitive labour market [16 p. 1055]. Randomised controlled trials confirmed that more than 60% of SE participants end up in long-term employment, compared to approximately 20–30% of other job seekers who have various forms of disabilities [17, 18]. SE has been described as ‘the most effective approach to labour inclusion’ [19 p. 74].

SE may be represented by SE models that target different population groups or/and use different follow-up techniques. The most researched and practiced SE models are the Individual Placement and Support (IPS) and Customized Employment (CE) models. IPS targets people with mental disorders. The goal is to provide participants with vocational placements in the competitive labour market as soon as possible after their start in the intervention [19, 20]. CE targets the same population as IPS but focuses on ‘exploratory time […] to uncover the job seeker’s unique needs, abilities, and interests.’ and the employer is expected to ‘voluntarily negotiat[e] specific job duties or employee expectations’ [21 p. 142].

During the last two decades, SE has been actively adopted by vocational rehabilitation agencies worldwide [22, 23]. Its target group has expanded to include veterans with traumatic brain injury [24, 25], people with chronic pain conditions, and, lately, people without mental or physical health issues but struggling to obtain a job in the competitive labour market, such as young adults who are Not in Education, Employment, or Training (NEET) and migrants, including refugees [26, 27].

The key to SE success appears to lie in its adopting of the person-centred approach that involves holistic non-directive counselling and implies that the participant is empowered to tailor the intervention according to his/her own needs and preferences by exercising self-determined informed choice, and including the participant in ‘service planning; respecting the person’s authenticity, self-determination, and choice … and facilitating engagement in the service’ [28 p. 4]; concurrently, the SE practitioner, often referred to as ‘employment specialist’ or ‘job coach’, provides non-directive counselling, facilitates collaborative engagement of the participant in the intervention, employing motivating and empowering techniques, for instance motivational interview (MI) [18, 28–30]. The participant is encouraged to make independent decisions while the counsellor plays mainly a supporting role in the person’s path to finding the authentic, right way to achieve his/her goals [31, 32]. Therefore, participant engagement seems to be the key to a proper person-centred intervention.

Within the SE literature, participant engagement is mostly mentioned in passing or as a recommendation in toolkits for practitioners (see for example, the European Union of Supported Employment Toolkit, European Union of Supported Employment [33]). In the EUSE toolkit, it is the first stage out of five stages of SE and implies the participant’s initial informed choice to participate in the SE and already on the recruitment stage exercises of empowerment and self-determination based on the information provided by the intervention providers. EUSE underlines that engagement is important over time during participation in the intervention, and the person-centred approach ensures that the engagement proceeds throughout all stages of the intervention.

Despite the importance of participant engagement in SE, there is no study that systematically focuses on participant engagement in SE. Neither is there a general definition of what participant engagement in SE is, with studies approaching this concept in different ways and from varying angles. Considering the importance of participant engagement in vocational rehabilitation interventions, the conceptualization of participant engagement in SE is a research gap that needs to be filled. The purpose of this study was to synthesise current research on participant engagement in SE; specifically, by answering the following research question: *How does the literature on SE present, define, and conceptualise participant engagement in SE interventions?* We aim to contribute to a broadly synthesised conceptualisation and definition of participant engagement in SE, which will facilitate its better understanding and implementation in SE and promote positive SE intervention outcomes.

## Methods

This systematic scoping review followed the steps recommended by Arksey and O'Malley [34] and the Preferred Reporting Items for Systematic Reviews and Meta-analyses guidelines, scoping review extension [35], completed in Appendix 1. A scoping review is a type of systematic review that systematically searches and identifies studies according to clearly defined inclusion and exclusion criteria, but rather than answering a specific research question regarding effect or experience, provides an overview of large research fields or fields that are not yet well-defined. In this case, the overall field of SE is a large and well-documented field; however, little is known about participant engagement in SE interventions.

### Search Strategy

For our systematic literature search, we developed a search strategy for the concept of ‘participant engagement’ for use in an SE context that was based on a 2017 review by Bonfils et al. on the implementation of the individual placement and support approach [36]. Considering that there is no generally accepted definition for the term and considering possible synonyms, based on the previous concept research in vocational rehabilitation (see Introduction), the search strategy included terms related to SE interventions, such as *supported employment*, *customised employment*, *individual placement and support*, and terms related to or synonymous with participant engagement in vocational intervention contexts, such as *engagement*, *empowerment*, *involvement*, *collaboration*, *working alliance*, and so on. We used truncation as appropriate. Appendix 2 contains the search strategy used in EBSCO.

**Fig. 1:**
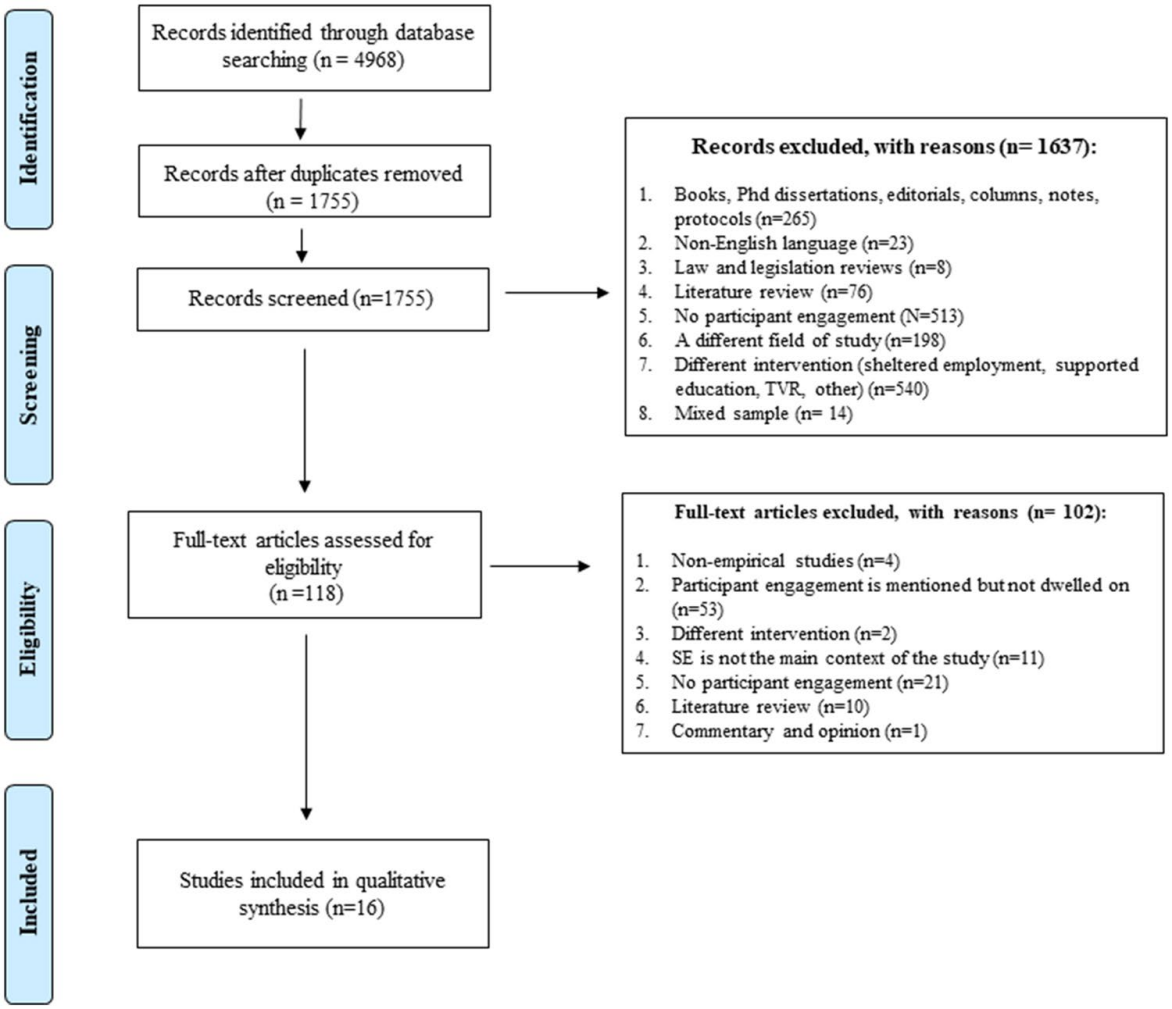
PRISMA flow diagram

The following databases were searched: EBSCO (Academic Search Premier, Academic Search Ultimate, ERIC, SocINDEX, CINAHL, PsycINFO, MEDLINE), SCOPUS, Social Care Online, and JSTOR. The search was completed on 9 October 2020. The search was not restricted by a date frame. The PRISMA flow diagram (Fig. 1) represents the processes of searching, screening, and retrieving articles.

### Selection Criteria and Data Extraction

We included peer-reviewed studies in English that provided empirical data and focused on SE targeting any populations, including groups with all types of disabilities and other support needs. Both qualitative and quantitative studies were included. Publications were excluded if the authors did not identify the studied intervention as SE, if SE was one of many studied interventions without a clear delineation of the interventions (mixed interventions), or if they were literature or legislation reviews. We also excluded articles that simply mentioned participant engagement but did not include any definition or explanation that was extractable.

In the first round of screening and selection, the titles and abstracts of 1755 publications were assessed by the first author (MK), as per the inclusion criteria. In total, 118 articles were assessed in full-text. The PRISMA flow diagram provides a full overview of study flow. Sixteen articles met the inclusion criteria and were included in the review.

### Analysis

Data analysis was conducted by the first author (MK) with QSR International’s NVivo v12 software. Data analysis was inspired by the thematic framework data analysis [37], which uses ‘a systematic process of sifting, charting and sorting material according to key issues and themes’ [37, p. 6]. The analysis and emergence of themes are based on previous research and involves the flexibility of the analyst and ability to ‘determine meaning, salience and connections’ [37, p. 6] of the analysed material and to identify newly emerging themes. The included articles were read several times, first, to familiarise ourselves with the content. Therewith, the themes, issues, and concepts relevant to the researched topic were highlighted, and the first thematic framework started to emerge. After all articles had undergone this process, in the next stage of analysis, repeating themes, concepts, or issues were placed into categories that were based partially on previous research on the topic of participant engagement in vocational rehabilitation (e.g. *self-determination, presence of options, motivation*) and which partially were new (e.g. *visualisation techniques, choice to defer, choice to retire*). The analysis and findings were presented to the review team and discussed to provide the most comprehensive interpretation to answer the research question. Finally, smaller categories were united into four main categories, which form the structure of the findings section.

## Results

Table 1 presents the characteristics of the 16 included studies, such as country, study location, aim, and design. The intervention overview, including target group and participants’ characteristics, is shown in Table 2.

**Table 1:** Study descriptions

Study	Country	Location	Study aim	Study methods/measures
Areberg and Bejerholm [42]	**Sweden**	Not specified	effectiveness of IPS in terms of occupational engagement, work-motivation, empowerment, and quality of life among people with SMI	Randomised controlled trial, interviews
Bejerholm and Björkman [41]	Sweden	Five outpatient centres in Malmoe	Describe and investigate empowerment and its relationship with level of engagement	Quantitative, cross-sectional study. A 28-item Empowerment Scale, Making Decisions; Manchester Short Assessment of Quality of Life; Profile of Occupational Engagement Scale; Rejection Experience Scale; Brief Psychiatric Rating Scale
Blankertz et al. [43]	USA	Two methadone treatment programs in New York City	Describe customised employment support, its principles, essential elements, and stages of service delivery	Description of the development of the intervention
Blankertz et al. [44]	USA	Two methadone treatment programs in New York City	Evaluate customised employment support in a randomised clinical trial	Description of the development of the intervention
Brady, Rosenberg, and Frain [46]	USA	Educational, rehabilitation, and employment settings across six geographic locations in Florida and Missouri	Present the role of the Job Observation and Behaviour Scale: Opportunity for Self-Determination scale in obtaining student and employee input into their own work performance and support needs	Quantitative, standardisation analyses of the Job Observation and Behaviour Scale: Opportunity for Self-Determination scale
Haslett et al. [40],	USA	Two sites in Chicago	Compare a computer tablet-based engagement intervention and a printed brochure for empowering participants to self-refer and engage in individual placement and support	Quantitative, randomised controlled trial
Johanson, Markström, and Bejerholm [48]	Sweden	Four mental healthcare services in the county of Skåne	to illustrate the IES model and process	Multiple-case design, Interviews, study of documentation and memos
Kilsby, Bennert, and Beyer [49]	UK	Two South Wales supported employment agencies	Focus on the problems of acquiescence in supported employment	Qualitative, discourse analysis of job review interviews
Kilsby and Beyer [50]	UK	Two South Wales supported employment agencies	Test two interventions aimed to increase self-determined vocational choices	Qualitative, direct observations and analysis of job review interviews
Kilsby and Beyer [58]	UK	Thirteen employment sites	Compare the interaction and engagement outcomes for supported employment and adult training centre participants	Qualitative, direct observation
Kostick, Whitley, and Bush [52]	USA	One community mental health hospital and two outpatient centres in Connecticut	Examine participant-centredness from the perspective of supported employment practitioners	Qualitative, semi-structured open-ended interviews
Larson et al. [47]	USA	Twenty-five mental health centres across the country	Investigate individual placement and support from a practitioner’s perspective	Quantitative, open-ended survey
McDermott and Edwards [39]	Australia	Thirty-one organisations across the country	Investigate what influences people’s decision to retire	Qualitative, in-depth qualitative interviews
Nittrouer, Shogren, and Pickens [38]	USA	A Midwest college town	Examine the impact of interventions derived from collaboration with person-centred teams and functional assessment of workplace problems	Qualitative, single-participant multiple baseline study observations
Solar [53]	Australia	Perth, Sir Charles Gairdner Hospital	Explore considering patient views in developing a linkage intervention	Qualitative, individual interviews
Wehmeyer et al. [45]	USA	Kansas	Examine the Girls at Work project with a focus on self-determination in vocational counselling	Intervention development description, Overview of an intervention model

**Table 2:** Descriptions of supported employment type and study participants

Study	Supported employment intervention type	Target group for intervention, in author(s)’ own words	Study participants		
			NN, role (intervention participants or practitioners)	Sex	Age (years)
Areberg and Bejerholm [42]	IPS	Participants with severe mental illnesses (exclusively)	120 participants in IPS, 2 in TVR	Not specified	20–65
Bejerholm and Björkman [41]	Not specified	People with mental illnesses (exclusively)	120 SE participants	Men (n = 67), women (n = 53)	Range = 21–58
Blankertz et al. [43]	CES	Methadone-maintained patients	NA	NA	NA
Blankertz et al. [44]	CES	Methadone-maintained patients	NA	NA	NA
Brady, Rosenberg, and Frain [46]	Not specified	Individuals with physical and mental disabilities	105 SE participants	Women (n = 47), men (n = 53)	Adult employees (n = 78), range = 22–67; high school students (n = 27), range = 17–21
Haslett et al. [40]	IPS	People with severe mental illnesses	45 IPS participants	NA	NA
Johanson, Markström, Bejerholm [48]	IES	People with affective disorders on long-term sleekness leave	5 participants, two employment specialists	Participants: 3 female, 2 male	25–52
Kilsby, Bennert, and Beyer [49]	Not specified	People with mental retardation	35 SE participants	NA	NA
Kilsby and Beyer [50]	Not specified	Job seekers with mental retardation	40 job seekers (5 dropped out or were not included in the final analysis) and 14 employment specialists	NA	Range = 19–52
Kilsby and Beyer [54]	Not specified	Individuals with learning disabilities	51 participants: 13 in SE and 38 in an ATC	SE: nine men and four women ATC: Twenty women and eighteen men	SE, range = 28–63 ATC: NA
Kostick, Whitley, and Bush [52]	IPS	People with severe mental illnesses	22 employment specialists	10 men	Mean age = 39.9
Larson et al. [47]	IPS	People with serious mental illnesses	67 SE practitioners	77% women	Mean age = 41.8
McDermott and Edwards [39]	Not specified	Older people with intellectual disabilities	Employees with a disability, n = 43; carers of employees, n = 2; supported employment providers, n = 12; supported employment providers (written submission), n = 14; other industry stakeholders, n = 5 (N = 76)	Employees: 60% men	Employees, range = 50–74; women < 65
Nittrouer, Shogren, Pickens [38]	CE	People with autism and/or intellectual disabilities	3 customised employment participants	1 woman, 2 men	Range = 22–29
Solar [53]	IPS	Patients with schizophrenia	20 IPS participants	12 women, 8 men	Range = 28–65
Wehmeyer et al. [45]	CE	Young women with intellectual and developmental disabilities	18 customised employment participants	All women	NA

*NA* not applicable, *SE* supported employment, *CES* customised employment support, *IPS* individual placement and support, *CE* customised employment, *ATC* adult training centre, *IES* individual Enabling and Support, *TVR* traditional vocational rehabilitation

### Description of the Included Studies

Most studies in the review were conducted in the USA (n = 8), followed by the UK (n = 3), Sweden (n = 3), and Australia (n = 2). The types of the included studies and their sample sizes varied. The most frequent methodological approach of the included studies was qualitative (n = 8), with the number of study participants ranging from three [38] to 76 [39]. In the quantitative studies (n = 5), the number of participants varied between 45 [40] and 120 [41, 42]. Three publications were descriptive and described the development of SE measures without specifying the data collection methods [43–45]. The SE interventions included CE (n = 2), IPS (n = 5), IES (1), and CES (n = 2), while six were not specified.

The intervention target groups were people with mental health disorders and/or learning disabilities that were either congenital or acquired owing to injuries or substance abuse. The authors described their participants as individuals with severe mental illnesses [41, 42], physical and mental disabilities [46], mental health issues [40, 47], affective disorders [48], mental retardation [49, 50], learning disabilities [51], methadone-maintained patients [43, 44], people with intellectual disability [39], severe mental disabilities [52], and intellectual and developmental disabilities [45]. Only a few studies provided a specific diagnosis: autism and/or intellectual disability [38] and chronic schizophrenia [53]. One study [46] included participants with both mental and physical disabilities.

As for participants’ demographic characteristics, only seven studies stated both participants’ sex and age or mean age group [38, 39, 41, 46, 53–55]. Bejerholm and Björkman [41] and Johanson et al. [48] provided the most comprehensive demographic characteristics, including ethnicity, age, sex, and family status. However, none of the studies provided an analysis of the collected data through the prism of demographic characteristics, even the studies that explored interventions among participants of a specific age [39, 46] or age and sex [45].

Most of the data presented in the studies focused on SE from the participants’ standpoint. Few articles included the standpoint of SE practitioners [39, 48, 50, 52], and only one article included other stakeholders [39].

### Conceptualisations of Participant Engagement

Three main themes emerged from the literature when conceptualising participant engagement in an intervention: expression of self-determined choice, collaboration/creating working alliance, and empowerment. The studies used only one of the themes or referred to several, and in this case, the three themes were often interrelated and complementary (Table 3).

**Table 3:** The three themes and connections between them

Theme	Sub-theme	Connected sub-themes
Expression of self-determined choice	Appropriate at different timepoints	Empowers participants from the very beginning of the intervention throughout the entire intervention Examples: [40, 52]
	Requirement of intervention success	Empowers the participant and fosters collaboration Example: [42]
	Consistency vs adjustability of choice	Achieved through collaboration Examples: [46, 49]
	Choice to stop participation	Empowered to make an uneasy but self-determined choice Examples: [39, 43, 44]
Collaboration/creating a working alliance	Provider communication	Facilitates understanding of the choices by the participant Examples: [46, 49]
	In decision-making	Availability of choices Examples: [45–47]
	Requirement for intervention success	A platform for participants to express their needs and choices and therefore, leads to better personalisation of intervention Examples: [46, 49]
	Role of service provider	Empowering to initiate collaborative process, providing choices Examples: [40, 44]
Empowerment	Role of service provider	Enabling self-determined choice Examples: [40, 47]
	Requirement for intervention success	Allows self-determined choice and collaboration, facilitates personalisation Examples: [42, 47]

#### Self-determined Choice

Expressing self-determination to participate in SE interventions and the exercise of self-determined choice selecting activities within the intervention was a main theme used to convey participant engagement by making a choice in the reviewed literature. Particularly, the availability of choices for SE participants seems to be a precondition to participant engagement, as participants engage through considering options and a self-determined choice that will influence further intervention direction and may lead to better intervention outcomes [45–47].

Kostick et al. [52] underline that the acknowledgement of participants’ employment choices and preferences and allowing them to direct the intervention accordingly is in accordance with the person-centred approach in SE, and determines if the participant succeeds or fails in his/her job. Limited availability and accessibility of jobs could result in reduced engagement [52]. Meanwhile, Kilsby and Beyer [50] argue that the choice should be consistent; that is, a participant should be encouraged not to change his/her choice, but to follow it through the intervention.

Expression of choice could be exercised at any stage, including pre-enrolment and exiting the intervention. While most studies underlined the importance of choice in employment-oriented activities during the intervention, Solar [53] emphasised the importance of SE intervention participants having the opportunity to make an initial choice to participate. Several studies brought into focus the importance of the choice to defer [43, 44] or retire [39] from an SE program. Retirement appeared as a sort of participant engagement, as this right to express self-determined choice is reportedly a difficult decision that participants often avoid initiating owing to its permanent nature and economic/social consequences. However, informing participants of the availability of this choice and helping them plan their life according to self-determined choice allows participants to ‘become self-determining beings’ [39 p. 431] and stay engaged in the intervention through decision-making until the very end.

#### Collaboration/Working Alliance

Several studies discussed the formation of a collaborative relationship between participants and SE practitioners, wherein both created a working alliance. Thus, Blankertz et al. [44] employed social psychological theory to argue that the creation of a working or therapeutic alliance based on a respectful and trusting collaborative relationship between the practitioner and the participant led to increased self-efficacy intervention benefits. They underlined the importance of participants trusting that the practitioner was genuine in his/her desire to help. They noted that such a genuine and trust-based relationship would help resolve participants’ previous experience of being rejected by society, which in their sample was related to their substance misuse.

Kilsby, Bennert, and Beyer [49] provided a different perspective on collaborative relationships in their study. They discussed interactions between participants and practitioners in terms of avoidance of acquiescence and enhanced self-determination. They underlined that acquiescence—participants’ passive confirmation of the options suggested and decided on by the SE practitioner—may appear as self-determined choice or collaboration, but is not, as the questions asked by practitioners in meetings with participants were mainly yes/no questions “requiring only minimal confirmation, and thus inviting acquiescent responses” [49] p. 296]. Therefore SE practitioners need to ensure that it is avoided by using open-ended questions in such meetings. The researchers focused on the way in which practitioners communicate with participants to ensure two-way collaboration and that the participants could express their self-determined choice despite their disabilities.

Brady, Rosenberg, and Frain [46] highlighted the importance of participant involvement in the decision-making process, noting that this offered the opportunity for self-determination and self-assessment techniques, while Larson et al. [47] underlined the importance of collaboration in problem solving and deemed it a success factor in SE interventions. They believed that collaboration is the foundation of a participant–practitioner relationship.

Finally, Nittrouer et al. [38] addressed collaboration in broader frames. They noted the importance of the participant’s collaboration with person-centred teams, which include vocational and treatment services, family members, and other supporting agencies.

#### Empowerment

While conceptualisations of participant engagement through making *self-determined choices* and *collaborating* are more focused on how engagement manifests through a participant’s actions, *empowerment* brings into focus the role of the service provider. Empowerment in the included studies appears as a process of engaging of the participant by the service provider, providing possibilities and facilitating independent actions and as a result, the participant being empowered to make self-determined choices [41, 42, 47]. Moreover, a study by Areberg and Bejerholm [42] pointed out that IPS participant empowerment and engagement are closely linked terms, while empowerment, choice, and working alliance are interrelated. IPS participants showed higher empowerment than those participating in traditional vocational rehabilitation (TVR); the authors suggested that this may have been due to the participant being empowered by making an informed choice and the attention to the participants’ preferences and collaboration with them on mapping out and following their choices. Moreover, they suggested that empowerment was related to the participants’ motivation and self-efficacy due to the constant focus of the intervention on the participants’ goals.

Haslett et al. [40] described empowerment as providing participants with accessible information on interventions and providing the means for participants to contact intervention providers using an online platform so that they could receive support when needed. They point out that empowering people through providing them with accessible information leads to their later self-determined choice to enrol in the intervention. Larson et al. [47] argued that empowerment implies participant-driven interventions: the practitioners’ focus on the participants’ independent choice increased their self-esteem. Focusing on the participant’s choices and strength and collaboration with the participant empowers the participant, facilitating his/her engagement (‘active participation’).

Bejerholm and Björkman [41] claimed that community integration and engagement in ‘daily activities and community life’ by the IPS participants were key aspects of empowerment, as these helped the participants to overcome the experience of stigma. The researchers underlined that SE was empowering as it increased individuals’ likelihood of obtaining employment, and therefore, being engaged in a meaningful activity and being integrated into the community. Such inclusion, in turn, improved participant engagement, both in the intervention and in community life.

Thus, empowerment seems to be an engaging process dependent on and initiated by the SE provider to engage the participant through providing him/her with information, choice, and creating a collaborative relationship with him/her. Moreover, empowerment seems to link collaboration/working alliance and self-determined choice together, as without a participant being empowered, the service will be provider-driven and the relationship between the provider and the participant can hardly be named a ‘working alliance’, wherein a participant can guide the service according to his/her own needs, choices, or preferences by making and incorporating self-determined choices.

## Discussion

This systematic scoping review is the first study to synthesise the available knowledge on participant engagement in SE interventions and answer the following research question: How does the literature on SE present, define, and conceptualise participant engagement in SE interventions?

Having analysed 16 articles, we found three themes used by the researchers to present participant engagement: exercise of self-determined choice, collaboration and the creation of a working alliance between the SE participant and employment specialists, and participant empowerment. The concepts of self-determined choice, empowerment, and collaboration are not synonymous but approach participant engagement from different angles, and they were often presented as inter-complementary and mutually reinforcing. This finding accords with previous research in rehabilitation counselling claiming that participant engagement in vocational rehabilitation is a construct combining motivation, empowerment, and a working alliance [9]. Without empowerment participants will not be able to exercise self-determined informed choice and actively participate in the intervention, such as career counselling [56]. In addition, Kosciulek and Wheaton [57] concluded that the empowering of intervention participants is a two-way process and involves the creation of a working alliance, participants’ informed choice, and their self-determination. According to our findings, participant engagement in SE is present if participants are empowered by the service providers and exercise self-determined choice, and there is a collaborative process, or working alliance, between the participants and SE practitioners.

The literature on participant engagement in vocational rehabilitation has suggested that there are two ways in which engagement takes place: a participant may be in a ‘state’ of being engaged or in a ‘process’ of becoming engaged [3]. In our opinion, a more dynamic definition of engagement as a *process* challenges the notion of conceptualising participant engagement as an ‘*end*-*state*’, as this does not bring into focus the practitioners’ role in empowering the participants and the importance of participants being engaged through choice and collaboration throughout the entire time that a person participates in a SE intervention. The literature we have reviewed supports the idea that engagement is more reasonable to see as a ‘process’, often generated, supported, and followed up on by a SE practitioner by empowering, creating, and maintaining the working alliance and providing the participant with choices, while the participant is being empowered, collaborates in the working alliance, and makes self-determined choices throughout the whole time that he/she is undergoing the intervention. We suggest, therefore, the following definition of participant engagement in SE: *Participant engagement in SE is an active, multifaceted process that involves the empowerment of participants, participants’ exercise of self-determined informed choice, and their collaboration with SE practitioners (employment specialists) in the working alliance.*

This systematic scoping review also revealed that the exact wording, *engagement*, though emerging in toolkits for SE practitioners, such as EUSE Toolkit, is a term that is seldomly used in the literature on SE consciously and consistently. Much of the literature employed different concepts and terms describing participant engagement in their interventions. This may lead to neglecting important parts of the process, as well as omitting factors that are important in ensuring that the SE intervention is aligned with person-centred fundamentals.

Meanwhile, the reviewed literature discussed participants’ engagement in work-oriented activities, treatment activities, daily life, and the community, pointing to a holistic person-centred approach that aims to improve the general functioning of an individual, his/her ‘improved coping with life’ [33 p. 69], as well as the formation of a ‘fully functioning person’ [33] where all the spheres of human life are equally important, including not only self-actualisation through meaningful activity, but also social belonging [33, 58]. Thus, the SE interventions were presented as interdisciplinary, targeting, as appropriate, the medical, employment, social, and other possible needs of the participants.

### Limitations

The systematic scoping review included peer-reviewed empirical studies of all study designs and captured a range of methodological approaches. We were therefore able to analyse conceptualisations of participant engagement that were described by intervention/trial investigators and by participants themselves. However, we did not search the grey literature, and doing so may have captured additional local or regional conceptualisations of participant engagement. Including only English-language studies also meant that findings from non-English speaking countries, without traditions of English-language publishing, were more likely to be excluded. Therefore, our analysis results may be less generalisable to countries that do not produce English-language academic works.

### Suggestions for Further Research

In all of the reviewed studies, only one, Blankertz et al. [44], employed a theoretical framework for their reasoning of participant engagement, in their study conveyed as the collaborating of SE practitioners and participants in a working alliance, with their reasoning originating in the relationship-building ideas of social psychology. The presence of a theoretical background in other studies would allow for more nuanced interpretations and for a more comprehensive understanding of the factors shaping participant engagement. We suggest that more studies employ a theoretical approach in research on participant engagement in SE. A theoretical framework would improve research validity by elucidating the participant engagement processes described in the reviewed studies. Particularly, we suggest the employment of the fundamentals of person-centred theory, including non-directive counselling and the development of a trust-based relationship, as such a reasoning of importance or exploration of participant engagement in SE would underline the person-centredness of SE and the processes behind participant engagement within a context of person-centred intervention. This would help stakeholders develop a comprehensive strategy for participant engagement implementation, utilisation, and development.

## Conclusion

This systematic scoping review has synthesised the available knowledge on participant engagement in SE interventions. Participant engagement in SE is a multidimensional concept that includes the empowerment of the intervention participants, their exercise of self-determined choice, and collaborating with SE practitioners in working alliances. This finding can guide the implementation, development, and practice of SE interventions by drawing attention to the empowering of the participants, ensuring that they are offered the self-determined choice possibility and collaborating in a working alliance. The finding can also be used by a broad range of rehabilitation services responsible for the vocational rehabilitation of people with various support needs.

## Supplementary Information

Below is the link to the electronic supplementary material.

Supplementary file1 (DOCX 107 kb)

Supplementary file2 (DOCX 12 kb)

## References

[1] Christle JW, Schlumberger A, Haller B, Gloeckl R, Halle M, Pressler A (2017). Individualized vs. group exercise in improving quality of life and physical activity in patients with cardiac disease and low exercise capacity: results from the DOPPELHERZ trial. Disabil Rehabil..

[2] Menchetti BM, Garcia LA (2003). Personal and employment outcomes of person-centered career planning. Educ Train Dev Disabil.

[3] Bright FAS, Kayes NM, Worrall L, McPherson KM (2015). A conceptual review of engagement in healthcare and rehabilitation. Disabil Rehabil.

[4] Williams MW, Rapport LJ, Hanks RA, Parker HA (2021). Engagement in rehabilitation therapy and functional outcomes among individuals with acquired brain injuries. Disabil Rehabil.

[5] King G, Chiarello LA, Ideishi R, Ziviani J, Phoenix M, McLarnon MJ, Pinto M, Thompson L, Smart E (2019). The complexities and synergies of engagement: an ethnographic study of engagement in outpatient pediatric rehabilitation sessions. Disabil Rehabil.

[6] Kubina LA, Dubouloz CJ, Davis CG, Kessler D, Egan MY (2013). The process of re-engagement in personally valued activities during the two years following stroke. Disabil Rehabil.

[7] Kabel A, McBee-Black K, Dimka J (2016). Apparel-related participation barriers: ability, adaptation and engagement. Disabil Rehabil.

[8] Waghorn G, Childs S, Hampton E, Gladman B, Greaves A, Bowman D (2012). Enhancing community mental health services through formal partnerships with supported employment providers. Am J Psychiatr Rehabil.

[9] Southwick JD, Schultz JC (2019). Participant engagement in public vocational rehabilitation programs: an analysis of counselor ratings. J Rehabil.

[10] Dutta A, Chan F, Kundu MM, Kaya C, Brooks J, Sánchez J, Tansey TN (2017). Assessing vocational rehabilitation engagement of people with disabilities: a factor-analytic approach. Rehabil Couns Bull.

[11] Iwanaga K, Chan F, Tansey TN, Strauser D, Ritter E, Bishop M, Brooks J (2019). Working alliance and stages of change for employment: the intermediary role of autonomous motivation, outcome expectancy and vocational rehabilitation engagement. J Occup Rehabil.

[12] Suhonen R, Stolt M, Charalambous A, Suhonen R, Stolt M, Papastavrou E (2019). Supporting individualised nursing care by leadership. Individualized care: theory, measurement and practice.

[13] Bishop M, Kayes N, McPherson K (2021). Understanding the therapeutic alliance in stroke rehabilitation. Disabil Rehabil.

[14] Johnson RL, Floyd M, Pilling D, Boyce MJ, Grove B, Secker J, Schneider J, Slade J (2009). Service users' perceptions of the effective ingredients in supported employment. J Ment Health.

[15] Cogan AM, Carlson M (2018). Deciphering participation: an interpretive synthesis of its meaning and application in rehabilitation. Disabil Rehabil.

[16] West M, Targett P, Wehman P, Cifu G, Davis J (2015). Separation from supported employment: a retrospective chart review study. Disabil Rehabil.

[17] Bond GR (2004). Supported employment: evidence for an evidence-based practice. Psychiatr Rehabil J.

[18] Hoffmann H, Jäckel D, Glauser S, Mueser KT, Kupper Z (2014). Long-term effectiveness of supported employment: 5-year follow-up of a randomized controlled trial. Am J Psychiatry.

[19] Nøkleby H, Hernes T (2017). Effekter av supported employment [The effects of supported employment]. Arbeid og velferd.

[20] Drake RE, Becker DR, Clark RE, Mueser KT (1999). Research on the individual placement and support model of supported employment. Psychiatr Q.

[21] Elinson L, Frey WD, Li T, Palan MA, Horne RL (2008). Evaluation of customized employment in building the capacity of the workforce development system. J Vocat Rehabil.

[22] Porteous N, Waghorn G (2007). Implementing evidence-based employment services in New Zealand for young adults with psychosis: progress during the first five years. Br J Occup Ther.

[23] Waghorn G, Hielscher E (2015). The availability of evidence-based practices in supported employment for Australians with severe and persistent mental illness. Aust Occup Ther J.

[24] Church HR, Seewald PM, Clark JM, Jak AJ, Twamley EW (2019). Predictors of work outcomes following supported employment in veterans with a history of traumatic brain injury. NeuroRehabilitation.

[25] Pogoda TK, Carlson KF, Gormley KE, Resnick SG (2018). Supported employment for veterans with traumatic brain injury: provider perspectives. Arch Phys Med Rehabil.

[26] Sveinsdottir V, Bull HC, Evensen S, Reme SE, Knutzen T, Lystad JU (2020). A short history of individual placement and support in Norway. Psychiatr Rehabil J.

[27] Maximova-Mentzoni T. Supported employment i kvalifiseringstiltak for innvandrere. To år med metodeutprøving og metodeutvikling i åtte forsøksprosjekter [Supported employment as a qualification measure for immigrants. Two years of method testing and eevelopment in eight pilot projects]. Arbeidsforskningsinstituttet, OsloMet – Oslo Metropolitan University (2019).

[28] Khoronzhevych M, Fadyl J (2020). How congruent is person-centred practice with labour activation policy? Person-centred approach to vocational interventions on immigrant jobseekers in Norway. Eur J Soc Work..

[29] Manthey TJ (2013). A pilot study of introductory motivational interviewing training for supported employment case managers. Int J Psychosoc Rehabil.

[30] Ståhl C, Gustavsson M (2018). Introducing motivational interviewing in a sickness insurance context: translation and implementation challenges. J Occup Rehabil.

[31] Hazler RJ, Capuzzi D, Gross DR (2007). Person-centered theory. Counseling and psychotherapy: theories and interventions.

[32] Rogers C (1967). On becoming a person.

[33] European Union of Supported Employment. European Union of Supported Employment Toolkit. 2010. https://www.euse.org/content/supported-employment-toolkit/EUSE-Toolkit-2010.pdf. Accessed 10 Apr 2018.

[34] Arksey H, O'Malley L (2005). Scoping studies: towards a methodological framework. Int J Soc Res Methodol.

[35] Prisma. Transparent Reporting of Systematic Reviews and Meta-Analyses. Extensions. 2015. http://www.prisma-statement.org/Extensions/ScopingReviews/. Accessed 15 Sept 2020.

[36] Bonfils IS, Hansen H, Dalum HS, Eplov LF (2017). Implementation of the individual placement and support approach – facilitators and barriers. Scand J Disabil Res.

[37] Ritchie J, Spencer L, Huberman AM, Miles MB (2002). Qualitative data analysis for applied policy research. The qualitative researcher’s companion.

[38] Nittrouer CL, Shogren KA, Pickens JL (2016). Using a collaborative process to develop goals and self-management interventions to support young adults with disabilities at work. Rehabil Res Policy Educ.

[39] McDermott S, Edwards R (2012). Enabling self-determination for older workers with intellectual disabilities in supported employment in Australia. J Appl Res Intellect Disabil.

[40] Haslett WR, McHugo GJ, Bond GR, Drake RE (2014). Use of software for tablet computers to promote engagement with supported employment: results from an RCT. Psychiatr Serv.

[41] Bejerholm U, Björkman T (2011). Empowerment in supported employment research and practice: Is it relevant?. Int J Soc Psychiatry.

[42] Areberg C, Bejerholm U (2013). The effect of IPS on participants' engagement, quality of life, empowerment, and motivation: a randomized controlled trial. Scand J Occup Ther.

[43] Blankertz L, Staines GL, Magura S, Madison EM, Horowitz E, Spinelli M, McKenzie A, Bali P, Guarino H, Grand A, Young R, Fong C (2003). The Customized Employment Supports (CES) model of vocational rehabilitation for methadone treatment patients. J Vocat Rehabil.

[44] Blankertz L, Magura S, Staines GL, Madison EM, Spinelli M, Horowitz E (2004). A new work placement model for unemployed methadone maintenance patients. Subst Use Misuse.

[45] Wehmeyer ML, Parent W, Lattimore J, Obremski S, Poston D, Rousso H (2009). Promoting self-determination and self-directed employment planning for young women with disabilities. J Soc Work Disabil Rehabil.

[46] Brady MP, Rosenberg H, Frain MP (2008). A self-evaluation instrument for work performance and support needs. Career Dev Except Individ.

[47] Larson JE, Sheehan L, Ryan C, Lemp S, Drandorff L (2014). Practitioner perspectives on Individual Placement and Support (IPS) for individuals with serious mental illness. J Vocat Rehabil.

[48] Johanson S, Markström U, Bejerholm U (2017). Enabling the return-to-work process among people with affective disorders: a multiple-case study. Scand J Occup Ther.

[49] Kilsby M, Bennert K, Beyer S (2002). Measuring and reducing acquiescence in vocational profiling procedures for first time job-seekers with mental retardation. J Vocat Rehabil.

[50] Kilsby MS, Beyer S (2002). Enhancing self-determination in job matching in supported employment for people with learning disabilities: an intervention study. J Vocat Rehabil.

[51] Kilsby MS, Beyer S (1996). Engagement and interaction: a comparison between supported employment and day service provision. J Intellect Disabil Res.

[52] Kostick KM, Whitley R, Bush PW (2010). Client-centeredness in supported employment: specialist and supervisor perspectives. J Ment Health.

[53] Solar A (2015). A Supported Employment linkage intervention for people with schizophrenia who want to work: a survey of patients' views. Australas Psychiatry.

[54] Kilsby M, Beyer S (1996). Engagement and interaction: a comparison between supported employment and ATCs. J Community Appl Soc Psychol.

[55] Johanson S, Markström U, Bejerholm U (2019). Enabling the return-to-work process among people with affective disorders: a multiple-case study. Scand J Occup Ther.

[56] Breeding RR (2008). Empowerment as a function of contextual self-understanding: the effect of work interest profiling on career decision self-efficacy and work locus of control. Rehabil Couns Bull.

[57] Kosciulek JF, Wheaton JE (2003). Rehabilitation counseling with individuals with disabilities: an empowerment framework [Editorial]. Rehabil Ed.

[58] McCormack B, McCance T (2010). Person-centred nursing: theory and practice.

