# Supplementary material for: Participant Engagement in Supported Employment: A Systematic Scoping Review
Source: J Occup Rehabil. 2021 Jun 4;32(3):414–25. doi: 10.1007/s10926-021-09987-2 (PMC9576634; doi:10.1007/s10926-021-09987-2)
Supplement: Supplementary file 2 — Supplementary file2 (DOCX 12 kb) [file 10926_2021_9987_MOESM2_ESM.docx]

Appendix 2

The search strategy used in EBSCO (Academic Search Premier, Academic Search Ultimate, ERIC, SocINDEX, CINAHL, PsycINFO, MEDLINE).

( “Support* employ*” OR “Customized Employment” OR “Individual Placement and Support” OR “supportive employment” OR “supporting employment” OR “customized support”) AND (“Engage*” OR “person-centered” OR “person-centred” OR “client-centered” OR “client-centred” OR “collaboration” OR “cooperation” OR “partnership” OR “alliance” OR “relationship” OR “motiv*” OR “self-determination” OR “choice” OR “choose” OR “empower*)”.
